# Periductal stromal sarcoma of the breast: A case report and review of the literature

**DOI:** 10.3892/ol.2014.2294

**Published:** 2014-06-27

**Authors:** YANDAN LAN, JIA ZHU, JIANLUN LIU, HUAWEI YANG, YI JIANG, WEI WEI

**Affiliations:** Department of Breast Surgery, The Affiliated Cancer Hospital of Guangxi Medical University, Nanning, Guangxi 530021, P.R. China

**Keywords:** periductal stromal sarcoma

## Abstract

Periductal stromal sarcoma (PSS), spindle and epithelioid types, is a rare subtype of malignant fibroepithelial tumor. The morphological characteristics of this neoplasm are different from phyllodes tumor and stromal sarcoma. PSS exhibits biphasic histology with benign ductal elements and a sarcomatous stroma composed of spindle cells and lacking phyllodes tumor architecture. The therapeutic management of PSS is based on wide surgery with free margins, and adjuvant therapies are not required. To the best of our knowledge, the recurrence of PSS in ≤5 months has not been reported in the literature to date. This report describes a 43-year-old woman who presented to our hospital with a recurrence of nodules in the left breast. The patient had undergone lumpectomy at a different hospital 5 months previously, and a diagnosis of phyllodes tumor was pathologically confirmed. On presentation at our hospital, the patient underwent a second lumpectomy. Histological examination revealed PSS and the patient underwent a simple mastectomy of the left breast with no adjuvant treatment (such as chemotherapy or radiotherapy). After 9 months of close follow-up examinations, no recurrence was observed. PSS is an extremely rare disease with low-grade sarcomatous behavior, which may evolve into a phyllodes tumor or an entity of breast cancer. Therefore, frequent follow-up examinations are required.

## Introduction

Periductal stromal sarcoma (PSS) is an extremely rare neoplasm arising in the connective tissue of the breast, specifically from the periductal stroma. PSS is a distinct, low-grade breast sarcoma with no clinical or radiological specificity. It has a biphasic morphology with benign ductal elements and a sarcomatous stroma lacking phyllodes tumor architecture, and thus is difficult to diagnose. The clinical presentation of a breast mass is non-specific and does not aid in the differentiation of this tumor from benign or malignant lesions. Similar to phyllodes tumors, PSS has a tendency for local recurrence when incompletely excised, and a potential to develop specific soft tissue sarcomas, as well as metastasis in cases harboring more aggressive sarcomatous patterns (1–5). Surgery with safe margins is the preferred therapeutic method for treating PSS, but the efficacy of adjuvant treatment (such as chemotherapy or radiotherapy) is yet to be clearly determined (1,2). Patient provided written informed consent.

## Case report

A 43-year-old woman, with no history of disease, presented to The Affiliated Cancer Hospital of Guangxi Medical University (Nanning, China) with a recurrence of nodules in the left breast. A pathological diagnosis of phyllodes tumor had been confirmed following lumpectomy at the People’s Hospital of Wuming (Nanning, China) 5 months previously. Upon presentation to our hospital, clinical examination identified a small mass measuring ~2.5 cm in diameter (ellipse in shape) in the upper outer quadrant of the left breast, and a large mass measuring ~5 cm in diameter (irregular shape) in the upper quadrant of the left breast adjacent to the areola with no signs of inflammation or association with axillary lymph nodes. Magnetic resonance imaging and molybdenum target computed radiography showed that the nodules of the left breast exhibited characteristics of phyllodes tumor or breast cancer; therefore, the patient underwent a core needle biopsy. Microscopic examination revealed mammary gland, acini and interstitial fibrous tissue hyperplasia, mild atypia and no obvious mitosis. Immunohistochemical analysis showed that the ductal epithelial cells were cytokeratin (CK)5/6-positive and less reactive for Ki-67 (~1% positive), while the stromal cells were ~20% positive for Ki-67. Comprehensive analysis of the patient’s medical history and immunohistochemical results suggested recurrent phyllodes tumor. Subsequently, the patient underwent lumpectomy of the left breast.

Grossly, the tumor of the upper quadrant of the left breast was a gray, solid and tenacious mass measuring 5.5×2.5×2 cm in size, with no significant hemorrhage or necrosis. The mass located in the upper quadrant of the left breast adjacent to the areola was part of the breast envelope, gray, solid and tenacious with no significant hemorrhage or necrosis, measuring 6.5×3.5×3 cm in size.

**Figure 1:**
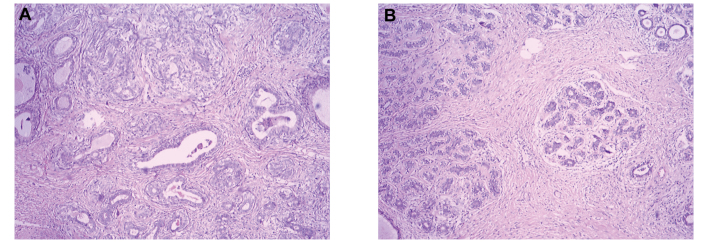
Microscopic examination of periductal stromal sarcoma revealed (A) epithelioid and spindle cells distributed in fibro-fatty tissue with no continuum boundary and (B) nodules and coalesced nodules, which are separated by fibro-fatty tissue (hematoxylin and eosin staining; magnification, ×40).

**Figure 2:**
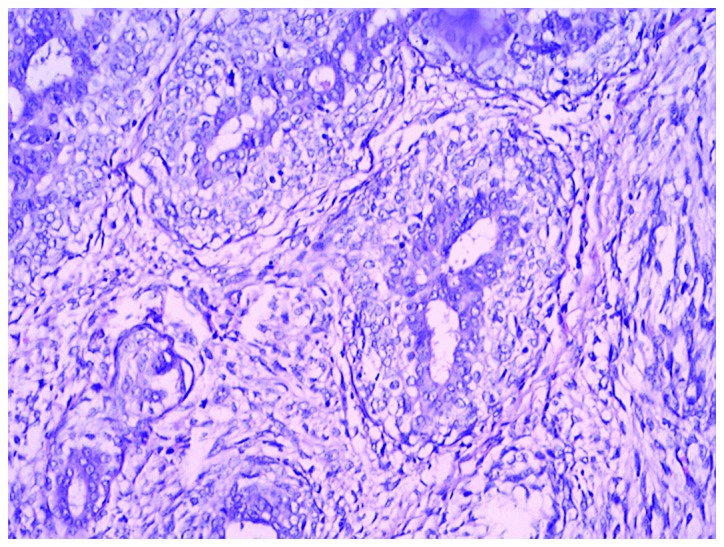
Cytology revealed epithelioid and spindle cells of periductal stromal sarcoma (hematoxylin and eosin staining; magnification, ×100).

**Figure 3:**
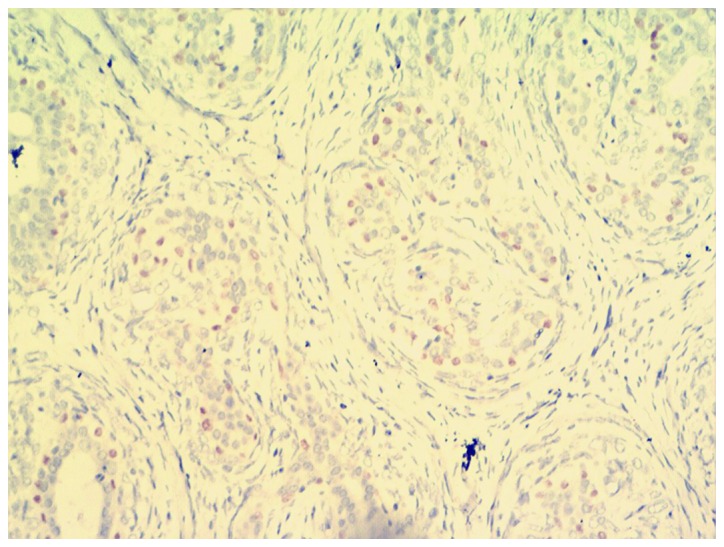
Periductal stromal sarcoma cells stained positive for cluster of differentiation 34.

Microscopic examination revealed no leafy architecture, however, biphasic proliferation composed of epithelial and mesenchymal components, predominantly polygonal (epithelioid) cells and spindle cell stromal proliferation around the epithelial (myoepithelial layer of ducts or ductules devoid of a phyllodes architecture) were observed, determining a diagnosis of PSS (low-grade, spindle and epithelioid types). The tissue sections were morphologically similar to those obtained during lumpectomy at the People’s Hospital of Wuming 5 months previously. More than 10 stromal mitotic figures per 10 high power fields were observed in the tissue from the second lumpectomy only (Figs. 1 and 2). Immunohistochemical analysis revealed that the glandular epithelial cells were CK19- and CK5/6-positive, the myoepithelial cells were smooth muscle antibody-, smooth muscle myosin heavy chain- and S-100-positive and glial fibrillary acidic protein-negative, and the tumor cells were cluster of differentiation (CD)34-positive and estrogen receptor (ER)- and progesterone receptor (PR)-negative (Fig. 3). Whether the resection margins were negative was not determined. The patient underwent a simple mastectomy of the left breast.

Microscopic examination revealed mesenchymal cell proliferation and no adjuvant treatment was administered. In a follow-up period of 9 months, the patient did not show any symptoms or signs of local or distant recurrence.

## Discussion

PSS was previously considered to be a variant of cystosarcoma with adipose metaplasia (1,3–5); however, currently, PSS is recognized as a separate entity and was classified by the World Health Organization in 2002 (6).

PSS occurs in perimenopausal and postmenopausal women (median age, 55.3 years) who are a decade older compared with those presenting with phyllodes tumor (median age, 45 years) (1). The common symptoms of PSS are similar to other benign and malignant breast tumors and have no radiological specificity (1). In the present case, the progress of PSS was similar to that of a malignant breast tumor and radiological examination led to a diagnosis of phyllodes tumor or breast cancer. A core needle biopsy and pathological examination were unable to conclude a diagnosis.

For middle-aged women presenting with breast lumps, clinicians must eliminate phyllodes tumor, which is an uncommon biphasic breast tumor with the ability to recur and metastasize and is biologically comparable to a stromal neoplasm. Similar to phyllodes tumor, PSS has a tendency for local recurrence when incompletely excised as well as a potential to develop specific soft tissue sarcomas and metastasize (1). Histologically, PSS is a biphasic breast tumor with benign ductal elements and a sarcomatous stroma lacking phyllodes tumor architecture (6). Thus, in this case, the patient was diagnosed with phyllodes tumor, following initial lumpectomy, which was then considered to have recurred after 5 months.

The histological characteristics of PSS were defined by the Armed Forces Institute of Pathology (AFIP) (1) as follows: i) A predominant spindle cell proliferation of variable cellularity and atypia around open tubules and ducts devoid of a phyllodes tumor pattern; ii) one or multiple nodules separated by adipose tissue; iii) stromal mitotic activity of ≥3/10 high-power fields; and iv) stromal infiltration into the surrounding breast tissue.

The histological grading depends on atypia and mitotic activity and ranges from low- to high-grade PSS (1). Immunohistochemically, PSS is CD34-positive and lacks S-100, ER and PR expression (6–9).

PSS is a tumor of intermediate behavior, resection with significant margins is generally considered sufficient and axillary lymphadenectomy is not required. With regard to adjuvant therapy, the currently available literature does not suggest any benefit of radiotherapy or chemotherapy. In the present case, the recurred nodules of the left breast following a lumpectomy 5 months before may be a result of resection performed without adequate margins. The patient underwent lumpectomy at our hospital, but as it was unclear whether the resection was performed with sufficient margins, the patient underwent further surgery. Microscopic examination of the breast tissue revealed mesenchymal cell proliferation; thus, the simple mastectomy of the left breast may have prevented future recurrence.

The tendency of PSS to recur and progress into phyllodes tumor or soft tissue sarcomas, as well as the occasional appearance of intraepithelial changes ranging from ordinary hyperplasia to intraductal carcinoma (1,2,9), indicate that close follow-up is required. The patient in the present case is currently recurrence-free 9 months after treatment.

In conclusion, the number of available studies on PSS is currently limited and a therapeutic strategy for PSS has yet to be determined. Histological diagnosis of PSS is based on the criteria established by the AFIP, and surgery with significant margins is the cornerstone of treatment. The prognosis of patients with PSS remains unclear; thus, increased experience of such cases and a longer follow-up period are required to investigate the optimal management and clinical behavior of this neoplasm.

## Abbreviations

PSS: periductal stromal sarcoma
